# 691. Infective Endocarditis with an Indication for Cardiac Surgery in a Tertiary Care Educational Hospital: Does Cardiac Surgery Improve Outcomes?

**DOI:** 10.1093/ofid/ofab466.888

**Published:** 2021-12-04

**Authors:** Deniz Akyol, Gunel Quliyeva, Selin Bardak özcem, Meral kayıkçıoğlu, Tansu Yamazhan, Sercan Ulusoy, Hilal Sipahi, Meltem Taşbakan, Oğuz Reşat Sipahi

**Affiliations:** 1 Doctor, İzmir, Izmir, Turkey; 2 Ege University Faculty of Medicine, Izmir, Turkey; 3 Professor Doctor, Izmir, Turkey; 4 Dr, Izmir, Turkey

## Abstract

**Background:**

In this retrospective cohort study, it was aimed to compare the clinical characteristics and outcomes of IE cases without and with an indication for cardiac surgery in terms of whether they have been operated or not, in a tertiary-care educational hospital.

**Methods:**

Patients that were followed up for definite IE (diagnosed according to modified Duke criteria between March 2007 and November 2020) with an indication for cardiac surgery according to European Society of Cardiology Guidelines, comprised the study group. Subjects were evaluated in terms of whether these cases have been operated or not, demographic features, underlying diseases, risk factors, clinical and laboratory findings, therapy responses, complications, and mortality. The timing of surgery is defined as emergency; surgery performed within 24 hours, urgent; within a few days, elective; after at least one-two weeks of antibiotic therapy. Statistical analysis was performed via Chi square and Student T tests and a p value < 0.05 was considered significant.

**Results:**

A total of 90 patients with an indication for surgery, 33.3% patients in underwent surgery, 66.6% patients in not underwent surgery group fulfilled the study criteria. The most frequently seen complaints in patients were fever (91.1%), cold-shiver (56.6%), weight-loss (27.7%), dyspnea (25.5%), and tachycardia (20%). Heart murmur was detected during cardiac auscultation of 44 patients. Mean blood leukocyte count, C-reactive protein and erythrocyte sedimentation rate were 12324 ± 6558/mm^3^ (1408-30330), 11.46 ± 8.38 mg/dl (0.18-34.6) and 61.43 ± 33.4 mm/h (2-130), respectively. There was no significant difference between two groups in terms of cardiac/non-cardiac risk factors, age, gender, etiologic agents, laboratory findings, septic embolisms and complaints (Table 1). In total IE with an indication for surgery mortality was 27.7%. Mortality rate was significantly less and heart murmur was significantly higher in cases who underwent surgery than those did not undergo surgery (p: 0.0447).

Table 1. Comparison of basic characteristics of patients in the two operated / unoperated cohorts.

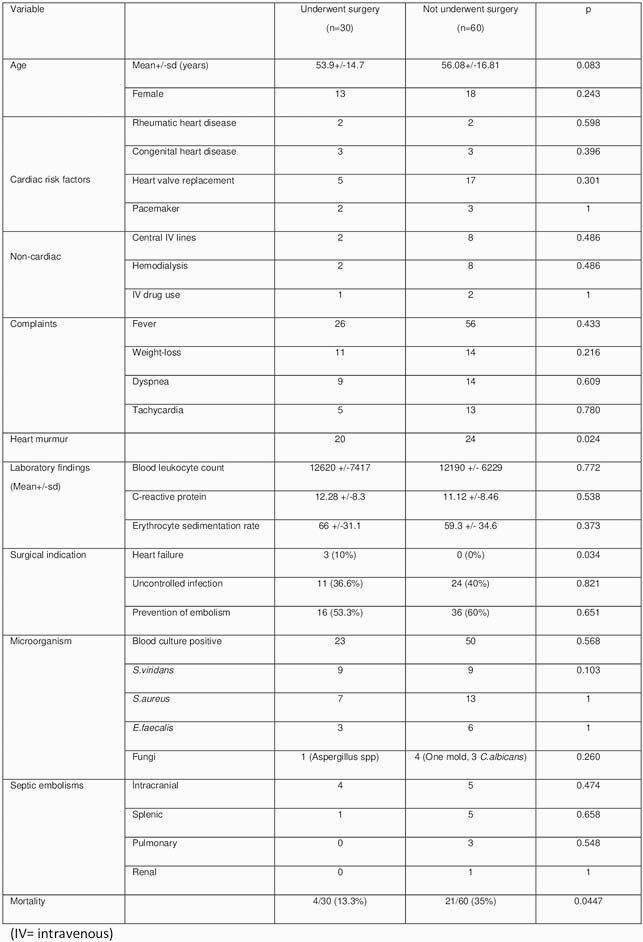

**Conclusion:**

These data support the importance of the guidelines’ criteria for cardiac surgery in the management of IE. Assuming that only 1/3 of the surgery needing cases received surgery, more interventions are needed to decrease the barriers against surgery.

**Disclosures:**

**All Authors**: No reported disclosures

